# Benign regulation of the gut microbiota: The possible mechanism through which the beneficial effects of manual acupuncture on cognitive ability and intestinal mucosal barrier function occur in APP/PS1 mice

**DOI:** 10.3389/fnins.2022.960026

**Published:** 2022-08-03

**Authors:** Xin Hao, Ning Ding, Yue Zhang, Yichen Yang, Yali Zhao, Jun Zhao, Yiran Li, Zhigang Li

**Affiliations:** ^1^School of Acupuncture, Moxibustion and Tuina, Beijing University of Chinese Medicine, Beijing, China; ^2^Guang’anmen Hospital, China Academy of Chinese Medical Sciences, Beijing, China; ^3^International School, Beijing University of Chinese Medicine, Beijing, China

**Keywords:** Alzheimer’s disease, manual acupuncture, gut microbiota, intestinal mucosal barrier, intestinal inflammation, probiotics

## Abstract

**Background:**

Gut microbiota dysbiosis and intestinal barrier injury play vital roles in Alzheimer’s disease (AD) onset and development. Our previous studies have demonstrated that manual acupuncture (MA) could improve the cognitive abilities of APP/PS1 mice. However, the effect of MA on the intestinal mucosal barrier and the gut microbiota mechanism through which this effect occurs remain to be clarified.

**Methods:**

In the APP/PS1 manual acupuncture (Am) group, MA was applied in Baihui (GV20), Yintang (GV29), and Zusanli (ST36). Mice in the APP/PS1 antibiotic + manual acupuncture (Aa) group were treated with an antibiotic mixture and MA at the same time. Probiotics were delivered to the APP/PS1 probiotics (Ap) group. Alterations in spatial learning and memory, the gut microbiota, the intestinal barrier function, and the expression of glial fibrillary acidic protein (GFAP), lipopolysaccharide (LPS), and TNF-α were evaluated in each group.

**Results:**

Compared with the C57BL/6 control (Cc) group, cognitive ability was significantly decreased, the gut microbiota structure was obviously disrupted, intestinal barrier integrity was drastically impaired, and the intestinal inflammatory response was enhanced in the APP/PS1 control (Ac) group (*P* < 0.01). These changes were reversed by MA and probiotics (*P* < 0.01 or *P* < 0.05), whereas antibiotics inhibited the benign regulation by MA (*P* < 0.01 or *P* < 0.05).

**Conclusion:**

Manual acupuncture can benignly modulate gut microbiota dysbiosis, significantly reduce intestinal inflammation, and effectively alleviate the destruction of the intestinal mucosal barrier in APP/PS1 mice, and the effects are comparable to those of probiotics. The gut microbiota may play an important role in the improvement of the cognitive function and intestinal barrier function by MA.

## Introduction

Alzheimer’s disease (AD) is a neurodegenerative disease and is the most common cause of dementia. AD is characterized by a progressive decline in several cognitive domains, including memory, language, executive and visuospatial functions, personality, and behavior, and is a major cause of disability and death (Weller and Budson, 2018; Alzheimer’s Dementia, 2020). According to a report, AD has become the fifth leading cause of death worldwide (Voulgaropoulou et al., 2019). At present, there are 40–50 million people suffering from AD in the world, and it is estimated that by 2050, the number of people with AD will reach 150 million (Qiu et al., 2009; Sherzai and Sherzai, 2019). The rapid increase in the number of AD patients poses a major challenge to public health and elderly care systems around the world and is a medical problem that requires urgent solutions.

Alzheimer’s disease can be hereditary, and the pathogenesis of AD is complicated (Pena-Bautista et al., 2019). Recent studies have indicated that the gut microbiota plays a key role in the pathogenesis of AD by regulating the brain function and behavior through the microbiota–gut–brain axis (Keshavarzian et al., 2015; Lai et al., 2018). In the development of AD, the steady state of gut microbiota is changed, mainly manifesting as a decrease in diversity and compositional changes characterized by an increase in the abundance of Gram-negative bacteria (Boren and Gershwin, 2004; Kumar et al., 2016). As a component of the Gram-negative bacterial cell wall, lipopolysaccharide (LPS) is a strong inducer of the inflammatory response (Zhang et al., 2009). The increase in intestinal Gram-negative bacteria causes the LPS concentration to be higher than normal (de J R De-Paula et al., 2018). Following LPS stimulation, the related inflammatory signaling pathways involving enteric glial cells (EGCs) are activated, and proinflammatory cytokines such as TNF-α and IL-1β are released, thus leading to the formation of an intestinal inflammatory environment (Cirillo et al., 2011). The intestinal inflammatory response affects the expression of intestinal tight junction proteins (occludin and ZO-1) and increases gut permeability (Zhang et al., 2009). The impairment of the intestinal mucosal barrier causes gut leakage, thereby allowing a large number of LPS, inflammatory factors, and other harmful metabolites to enter the circulatory system and inducing a low-grade systemic inflammatory response (Cani et al., 2012; Moreno-Navarrete et al., 2012). The upregulation of inflammatory cytokines damages the blood–brain barrier, allowing these cytokines to flow into the brain through the blood, thus activating microglia. Persistent activation of microglia can trigger a series of immune cascade reactions, thus causing neuroinflammation and neurodegeneration, which eventually lead to the development of AD (Cattaneo et al., 2017; Fawley et al., 2017; Dumitrescu et al., 2018; Wang et al., 2018). Therefore, the impairment of the intestinal mucosal barrier function is the key link in a series of pathological cascades of AD induced by gut microbiota dysbiosis (Goyal et al., 2021). The increase in intestinal Gram-negative bacteria and the resulting LPS exposure can induce intestinal inflammation, which is an important mechanism for the destruction of the intestinal mucosal barrier (Sommer et al., 2017; Bhattarai, 2018).

Our previous work confirmed that manual acupuncture (MA) plays an important role in the treatment of AD (WuLi et al., 2021). MA can effectively improve the cognitive function and delay the pathological process of AD through the effects of anti-inflammation (Jiang et al., 2018; Ynag et al., 2021), the regulation of energy metabolism (Liu et al., 2017; Xu et al., 2020), and the regulation of cerebral blood flow (Ding et al., 2019a,b). Recently, the correlation between the microbiota–gut–brain axis and the pathogenesis and development of AD has become gradually clear, becoming a new target for the prevention and treatment of AD. Numerous studies have verified that MA can effectively treat a variety of diseases by benignly regulating the gut microbiota and preserving intestinal barrier integrity (Savari et al., 2014; Ma et al., 2019; Liu G. H. et al., 2020). However, the effect of MA on the intestinal mucosal barrier of APP/PS1 mice has not yet been reported, and the mechanism by which MA improves the cognitive function by affecting the gut microbiota remains to be clarified. In view of this, we observed the influences of MA on the spatial learning and memory ability, gut microbiota, intestinal inflammation, and intestinal mucosal barrier of APP/PS1 mice, and we explored the gut microbiota mechanism by which MA regulates intestinal mucosal barrier function. The findings of this study will contribute to clarifying the intestinal mechanism of MA in the treatment of AD and promoting the popularization and spread of MA in AD treatment.

## Materials and methods

### Experimental animals

Male APP/PS1 mice and male C57BL/6 mice were purchased from Cavens Biogle (Suzhou) Model Animal Research Co. Ltd. and tested by Suzhou Xishan Biotechnology INC (Animal Lot: SCXK (Su) 2018-0002). Both types of mice weighed 30.0 ± 2.0 g and were 6 months old. The animals were housed in the Experimental Animal Center of the Beijing University of Chinese Medicine at a controlled temperature (24 ± 2°C) and under a 12-h dark/light cycle, with sterile drinking water and a standard pellet diet available *ad libitum*. All mice were acclimatized to the environment for 7 days prior to experimentation, and all experimental procedures complied with the ARRIVE guidelines and were performed according to the guidelines of the National Institutes for Animal Research (ID: bucm-4-2021102701-4032).

### Animal grouping and intervention

A total of 18 C57BL/6 mice were used as the control (Cc) group, and 72 APP/PS1 mice were divided into four groups (*n* = 18 per group): the APP/PS1 control (Ac) group, the APP/PS1 manual acupuncture (Am) group, the APP/PS1 antibiotic + manual acupuncture (Aa) group, and the APP/PS1 probiotics (Ap) group.

The mice in the Am group were immobilized in mouse bags. Disposable sterile acupuncture needles (0.25 mm × 13 mm) (Beijing Zhongyan Taihe Medicine Company, Ltd.) were used. MA at Baihui (GV20), Yintang (GV29), and Zusanli (ST36) was applied for 20 min once a day from days 8 to 45, with transverse puncturing at a depth of 3 mm (Baihui and Yintang) and perpendicular puncturing at a depth of 4 mm (Zusanli). The selection and position of the acupoints and acupuncture method were based on findings of our previous studies (Liu et al., 2010; Jiang et al., 2015; Cao et al., 2017). For the Aa group, the antibiotic mixture (containing ampicillin 50 mg/kg, neomycin 60 mg/kg, vancomycin 25 mg/kg, metronidazole 60 mg/kg, and clindamycin 150 mg/kg) was delivered to the mice by oral gavage once a day from days 1 to 7 (Hintze et al., 2014; Staley et al., 2017; Guida et al., 2018; Zeng et al., 2020). Then, from days 8 to 45, the mice were given antibiotic water (containing ampicillin 1.0 mg/ml, neomycin 0.5 mg/ml, vancomycin 0.5 mg/ml, metronidazole 1 mg/ml, and clindamycin 0.5 mg/ml) (Lamouse-Smith et al., 2011; Minter et al., 2016) and MA at the same time. The selection and position of the acupoints and acupuncture method were identical to those in the Am group. The mice in the Ap group were administered probiotics (8.7 × 10^8^ CFU/g/day, containing *Bifidobacterium animalis* ssp. *lactis HN019, Bifidobacterium bifidum Bb06, Bifidobacterium animalis* ssp. *lactis BB-12, Bifidobacterium animalis* ssp. *lactis Bi07, Bifidobacterium longum R175, Bifidobacterium animalis B94, Lactobacillus rhamnosus GG, L. casei Lc11, Lactobacillus helveticus R52, Lactobacillus paracasei Lpe37, Lactobacillus plantarum R1012, Lactobacillus reuteri HA188, Lactobacillus rhamnosus R11, Lactobacillus acidophilus NCFM*, and *Streptococcus thermophiles St21*) (Beijing Zhongke Yikang Biotechnology Company, Ltd.) by oral gavage once a day from days 8 to 45 (Ni et al., 2019). The mice in the Cc and Ac groups were immobilized for 20 min in the same manner as the mice in the Am group from days 8 to 45.

### Animal disposing and sample collection

On days 1 and 7, we randomly selected eight mice from the Aa group and collected fresh fecal tissues. On day 46, fresh fecal tissues were gathered from eight mice in each group. These samples were used for the 16S rRNA sequencing. From days 40 to 45, 10 mice in each group were selected for the Morris water maze (MWM) test. On day 46, all the mice were anesthetized by an intraperitoneal injection of pentobarbital (80 mg/kg body weight), and the samples were collected for the laboratory test: (1) six mice from each group were chosen for immunofluorescence (IF) staining; (2) six mice from each group were subjected to intestinal permeability assessment, and then their small intestinal tissues were harvested and used for hematoxylin and eosin (HE) staining and transmission electron microscopy (TEM) analysis (from three mice); (3) six mice from each group were subjected to Western blot (WB) and enzyme-linked immunosorbent assay (ELISA) analysis.

### Morris water maze test

A total of 10 mice in each group were subjected to the hidden platform trial and the probe trial in turn. The MWM test we used in this study has been described previously (Ding et al., 2019b). The platform was located in the middle of the southwest (SW) quadrant. Each mouse was released from one of four start locations and had 60 s to search for the hidden platform. At the end of each trial, the mouse was placed on the platform or allowed to stay there for 15 s. The escape latency and swimming speed were collected for subsequent analysis. The hidden platform trial was performed for five consecutive days. After the hidden platform trial, the platform was removed, and each mouse was placed in the pool once for 60 s. The starting direction farthest from the platform quadrant was used in the probe trial. The platform crossover number and swimming trace were recorded. The probe trial was performed for 1 day.

### 16S rRNA sequencing

Fecal microbiota composition was identified by 16S rRNA as described previously (Xue et al., 2020). Microbial community genomic DNA was extracted by using the E.Z.N.A.^®^ soil DNA Kit (Omega Bio-tek, Norcross, GA, United States). The DNA concentration and purity were checked, and the 16S rRNA gene library preparation was conducted by using Polymerase chain reaction (PCR) amplification of the V3–V4 region. All libraries were sequenced using the Illumina MiSeq PE300 platform/NovaSeq PE250 platform (Illumina, San Diego, CA, United States) according to the standard protocols by Majorbio Bio-Pharm Technology Co. Ltd. (Shanghai, China). Bioinformatics analyses were performed to compare the differences in the gut microbiota between the experimental groups.

### Immunofluorescence staining

Small intestine tissues were fixed with paraformaldehyde and sectioned by using a freezing microtome (CM1900, Leica Corporation, Germany). The sections were washed and then blocked. Next, the sections were treated with the mouse monoclonal glial fibrillary acidic protein (GFAP) antibody (1:100, Abcam, United States), rabbit polyclonal occludin antibody (1:100, Invitrogen, United States), rabbit polyclonal ZO-1 antibody (1:100, Invitrogen, United States), and mouse polyclonal lipopolysaccharide core antibody (1:100, Hycult Biotech, United States), respectively, and incubated overnight. After washed, the sections were exposed to respective secondary antibodies, including donkey anti-mouse IgG Alexa Fluor 594 (1:100, Abcam, United States) and donkey anti-rabbit IgG Alexa Fluor 488 (1:100, Abcam, United States). Ultimately, the sections were stained with DAPI (DAPI, Abcam, Netherlands). Observations were performed under a confocal laser scanning microscope (SP8, Leica, United States).

Identical exposure times and image settings were used for each experiment. The specimens treated with GFAP, LPS, occludin, and ZO-1 were captured at 100× and 63× magnification for quantification, respectively. Each sample randomly selected three fields of view for detecting (Cerovic et al., 2019; Wang et al., 2019; Kumar et al., 2021). The number of cells expressing GFAP and the mean optical density of occludin, ZO-1, and LPS were analyzed using ImageJ.

### Intestinal permeability assessment

The mice were given 4KD FITC-dextran (Sigma-Aldrich, St. Louis, MO, United States) by oral gavage (0.6 mg/g) as previously described (Cani et al., 2008). After 4 h, blood was taken from the eyeball and centrifuged. Then, the supernatant was collected. Concentrations of FITC were determined in 100 μl serum samples by using molecular devices with an excitation of 485 nm and an emission of 535 nm.

### Hematoxylin and eosin analysis

The small intestine tissues were fixed with paraformaldehyde and embedded in paraffin. Then, the tissues sectioned and stained using standard protocols. Identical exposure times and image settings were used for each experiment. The epithelial morphological characteristics were observed microscopically (Leica, United States) at 20× magnification. Chiu’s scores were determined under blinded conditions using a histologic injury scale as previously describe (Chiu et al., 1970).

### Transmission electron microscopy analysis

Small intestine tissues were fixed with 2.5% glutaraldehyde and osmium tetroxide buffer separately and then dehydrated and embedded. The samples were then cut into ultrathin sections and stained with uranyl acetate and lead citrate. Epithelial intestinal tight junctions were observed using a TEM (FEI TECNAI SPIRIT) at 16,500× magnification.

### Western blot analysis

After homogenate and protein extraction, SDS-PAGE was performed with a 8% separating gel and a 5% stacking gel and transferred to a 0.45-μm PVDF membrane. Then, the membranes were incubated with occludin (1:500, Invitrogen, United States), ZO-1 (1:500, Invitrogen, United States), and beta-actin (1:500, Bioss, United States). After washing, the proteins of interest were labeled with the secondary antibody (goat polyclonal rabbit IgG antibody-HRP, 1:3000, Bioss, United States). HRP-ECL luminous liquid was added, and X-ray film exposure was completed in a dark room following development and fixation. All WB bands were normalized with their corresponding β-actin expression for the appropriate evaluation of protein expressions. The relative expression of occludin and ZO-1 was compared in each group.

### Enzyme-linked immunosorbent assay analysis

After centrifuging the blood and homogenizing of the small intestine tissues, the supernatant was collected, respectively. The concentration of TNF-α in the plasma and intestine was determined using ELISA kits (Oubei Biotechnological Co. Ltd., Beijing).

### Statistical analysis

Statistical analysis was performed by SPSS 25 software. The data are expressed as mean ± standard deviation. A two-way analysis of variance (ANOVA) with repeated measures was used to analyze group differences in the hidden platform trial. A one-way ANOVA, followed by the LSD multiple-range test, was used to analyze group differences in other experiments. For the non-normally distributed data or data with heterogeneous variance, the Kruskal–Wallis test was used. The statistical significance was set to *P* < 0.05, and a high statistical significance was set to *P* < 0.01.

## Results

### Effects of manual acupuncture on spatial learning and memory

**FIGURE 1:**
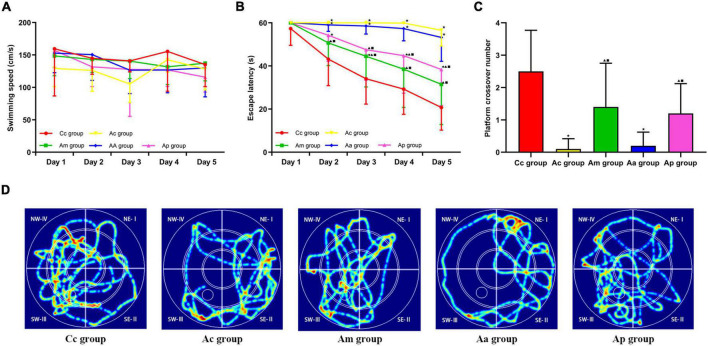
Results of the Morris water maze (MWM) tests in each group (*n* = 10, mean ± SD). **(A,B)** Comparison between the swimming speed and escape latency of all groups in the hidden platform trial. **(C)** Comparison between the platform crossover numbers of all groups in the probe trial. A two-way analysis of variance (ANOVA) with repeated measures, followed by the LSD multiple-range test, was used with an exception when comparing the platform crossover numbers, which was analyzed by using the Kruskal–Wallis test. LSD-t and chi-square are presented in Supplementary Tables 1, 2. ^★^*P* < 0.01 or *P* < 0.05 compared with the C57BL/6 control (Cc) group, ^▲^*P* < 0.01 or *P* < 0.05 compared with the APP/PS1 control (Ac) group, ^■^P < 0.01 or *P* < 0.05 compared with the APP/PS1 antibiotic + manual acupuncture (Aa) group. **(D)** Swimming trace of all groups.

The results of the MWM test are presented in Figure 1. In the hidden platform trial, there was no significant difference in the escape latency among groups on day 1. The escape latencies of the Cc, Am, and Ap groups decreased gradually, while the escape latencies of the Ac and Aa groups remained high. From days 2 to 5, the escape latency of the Ac group was significantly higher than that of the Cc group (*P* < 0.01). The escape latencies of the Am and Ap groups were drastically lower than those of the Ac group on days 2–5 and days 3–5, respectively (*P* < 0.01 or *P* < 0.05), but were still higher than those of the Cc group (*P* < 0.01). The escape latency of the Aa group was drastically higher than that of the Am and Cc groups on days 2–5 and was drastically higher than that of the Ap group on days 3–5 (*P* < 0.01 or *P* < 0.05). There was no significant difference in swimming speed among groups.

In the probe trial, the platform crossover number of the Ac group was drastically lower than that of the Cc group (*P* < 0.01). The platform crossover numbers of the Am and Ap groups were significantly higher than those of the Ac group (*P* < 0.05). In the Aa group, the platform crossover number was drastically lower than that in the Cc, Am, and Ap groups (*P* < 0.01 or *P* < 0.05). The swimming traces in the Cc, Am, and Ap groups all showed a tendency of swimming strategy, mainly toward the SW quadrants. However, in the Ac and Aa groups, the traces were mostly random or marginal.

### Effects of manual acupuncture on gut microbiota diversity and composition

**FIGURE 2:**
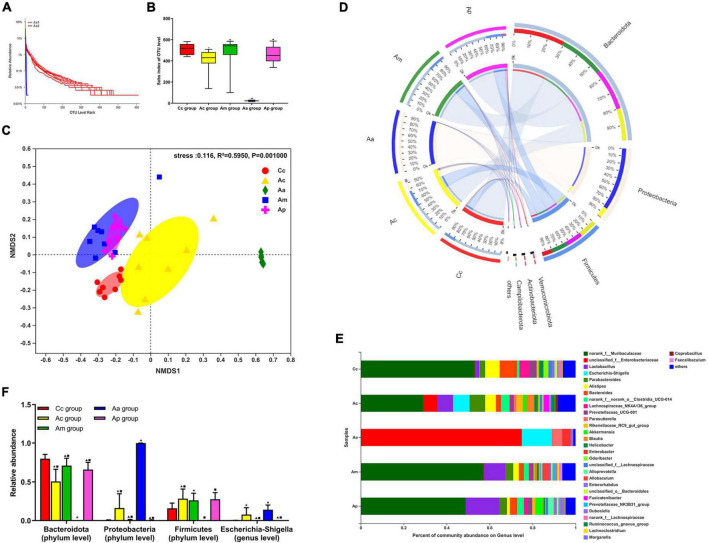
Results of the 16S rRNA in each group (*n* = 10, mean ± SD). **(A)** Rank abundance curves in the APP/PS1 antibiotic + manual acupuncture (Aa) group on day 1 (before antibiotic pretreatment, Aa 1) and day 7 (after antibiotic pretreatment, Aa 2). **(B)** α-Diversity analysis of fecal microbiota by the Sobs index. **(C)** β-Diversity analysis of fecal microbiota by NMDS. **(D–F)** Microbial relative abundance at the phylum and genus levels. The Kruskal–Wallis test was used. Chi-square was presented in Supplementary Tables 2, 3 ^★^*P* < 0.01 or *P* < 0.05 compared with the C57BL/6 control (Cc) group, ^▲^*P* < 0.01 or *P* < 0.05 compared with the APP/PS1 control (Ac) group, ^■^*P* < 0.01 or *P* < 0.05 compared with the APP/PS1 antibiotic + manual acupuncture (Aa) group.

The results of the 16S rRNA are presented in Figure 2. Rank abundance curves showed that the Aa 1 curve had a wide distribution range and a gentle shape on the horizontal axis, while the Aa 2 curve had a narrow distribution range and suddenly dropped on the horizontal axis. In the α-diversity analysis, the Sobs index of the Ac and Aa groups was significantly lower than that of the Cc group (*P* < 0.01 or *P* < 0.05). Compared with the Aa group, the Sobs index in the Ac, Am, and Ap groups increased markedly (*P* < 0.01), whereas there were no significant differences in the Sobs index among the Ac, Am, and Ap groups. In the β-diversity analysis, there was a clear separation of the Ac group from the Cc, Am, and Ap groups. Additionally, the Aa group formed a unique cluster that was separate from the other groups. However, there was an obvious clustering between the Am group and the Ap group.

The community composition analysis revealed the differences in taxonomic abundance between different groups. At the phylum level, Bacteroidetes, Proteobacteria, and Firmicutes were the three most abundant bacteria among all groups. At the genus level, Muribaculaceae, Enterobacteriaceae, *Lactobacillus*, and *Escherichia-Shigella* were the four most abundant bacteria among all groups. Compared with the Cc, Am, and Ap groups, the abundance of Bacteroidetes in the Ac group was markedly reduced (*P* < 0.01 or *P* < 0.05), while the abundance of Proteobacteria and *Escherichia-Shigella* was significantly increased (*P* < 0.01), and the abundance of Firmicutes in the Ac group was significantly higher than that of the Cc group (*P* < 0.05). Compared with the Cc, Ac, Am, and Ap groups, the abundance of Firmicutes and Bacteroidetes in the Aa group was drastically decreased (*P* < 0.01), the abundance of Proteobacteria was significantly increased (*P* < 0.01), and the abundance of *Escherichia-Shigella* in the Aa group was significantly higher than that in the Cc, Am, and Ap groups (*P* < 0.01 or *P* < 0.05).

### Effects of manual acupuncture on the intestinal mucosal barrier

**FIGURE 3:**
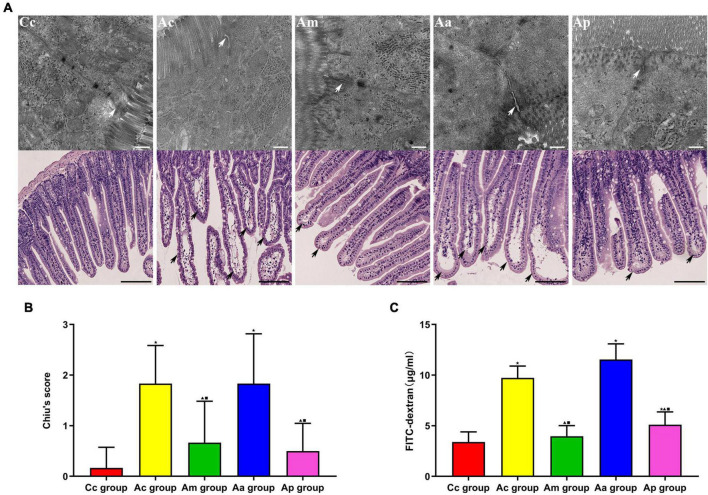
Evaluations of the intestinal barrier integrity and function in each group (*n* = 6, mean ± SD). **(A)** Representative images of hematoxylin and eosin (HE) staining (scale bar = 100 μm) and Transmission electron microscopy (TEM) (scale bar = 500 μm) in each group. White arrows show the tight junctions (TJs) between intestinal epithelial cells, and black arrows show intestinal mucous injury sites. **(B)** Comparison between Chiu’s score of all groups. **(C)** Comparison between the FITC-dextran level of all groups. A one-way analysis of variance (ANOVA), followed by the LSD multiple-range test, was used with an exception when comparing Chiu’s score, which was analyzed by the Kruskal–Wallis test. LSD-t and chi-square are presented in Supplementary Tables 2, 4. ^★^*P* < 0.01 or *P* < 0.05 compared with the C57BL/6 control (Cc) group, ^▲^*P* < 0.01 or *P* < 0.05 compared with the APP/PS1 control (Ac) group, ^■^*P* < 0.01 or *P* < 0.05 compared with the APP/PS1 antibiotic + manual acupuncture (Aa) group.

The evaluations of intestinal barrier integrity and function are presented in Figure 3. In the Cc group, small intestinal villi were normal and complete in morphology, with clear borders and no edema or blunting. The tight junctions (TJs) between epithelial cells were intact and compact. In the Ac and Aa groups, the apical epithelial gap of small intestinal villi was enlarged, the epithelial layer was moderately separated from the lamina propria, and the tips of the villi were broken. Damaged TJ structures with loosened connections and widened gaps were observed. The damage to the structure of the small intestine in the Am and Ap groups was significantly reduced, manifesting as mild separation of the epithelial layer from the lamina propria, a more orderly arrangement of intestinal gaps and narrower connection gaps.

Chiu’s score and FITC-dextran levels in the Ac and Aa groups were significantly higher than those in the Cc group (*P* < 0.01). Markedly lower Chiu’s scores and FITC-dextran levels were found in the Am and Ap groups than in the Ac and Aa groups (*P* < 0.01 or *P* < 0.05).

### Effects of manual acupuncture on the expression of tight junction proteins (occludin and ZO-1) in the intestine

**FIGURE 4:**
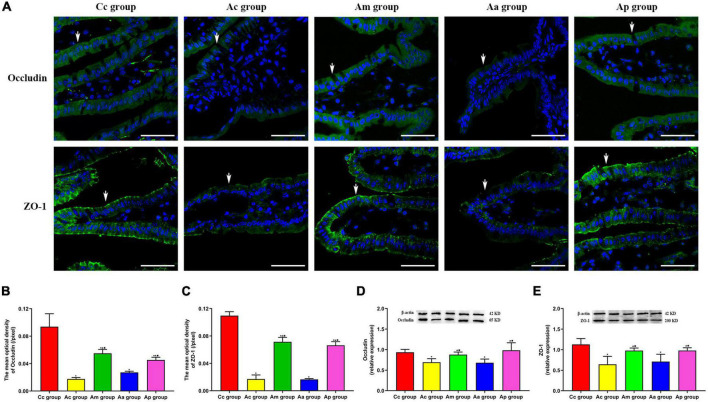
Results of the expression of intestinal tight junction proteins occludin and ZO-1 in each group (*n* = 6, mean ± SD). **(A)** Representative images of immunofluorescence (IF) staining of occludin (green) and ZO-1 (green) in each group, the positively stained cells are shown with white arrows, and scale bar is 50 μm. **(B,C)** Comparison between the mean optical density of occludin and ZO-1 of all groups. **(D,E)** Comparison between the relative expression of occludin and ZO-1 of all groups. A one-way analysis of variance (ANOVA), followed by the LSD multiple-range test, was used with an exception when comparing the relative expression of ZO-1, which was analyzed by the Kruskal–Wallis test. LSD-t and chi-square are presented in Supplementary Tables 5–7. ^★^*P* < 0.01 or *P* < 0.05 compared with the C57BL/6 control (Cc) group, ^▲^*P* < 0.01 or *P* < 0.05 compared with the APP/PS1 control (Ac) group, ^■^*P* < 0.01 or *P* < 0.05 compared with the APP/PS1 antibiotic + manual acupuncture (Aa) group.

The results of the expression of TJ proteins (occludin and ZO-1) in the intestine are presented in Figure 4. TJ proteins are mainly distributed on the cell membrane between adjacent cells in the apical part of the intestinal epithelium, with a continuous distribution, and tight intercellular junctions. The fluorescence structure of occludin and ZO-1 in the Cc group was clear, with strong continuity and neat arrangement. Compared with the Cc group, the fluorescence structure of occludin and ZO-1 in the Ac and Aa groups was disordered and weakened. The fluorescence structure of occludin and ZO-1 in the Am and Ap groups was restored, with continuity and enhanced intensity.

The mean optical density and relative expression of occludin and ZO-1 in the Ac and Aa groups were all significantly lower than those in the Cc group (*P* < 0.01). Compared with the Ac and Aa groups, the mean optical density and relative expression of occludin and ZO-1 in the Am and Ap groups were all increased markedly (*P* < 0.01).

### Effects of manual acupuncture on the number of cells expressing glial fibrillary acidic protein and the expression of lipopolysaccharide and TNF-α in serum and intestine

**FIGURE 5:**
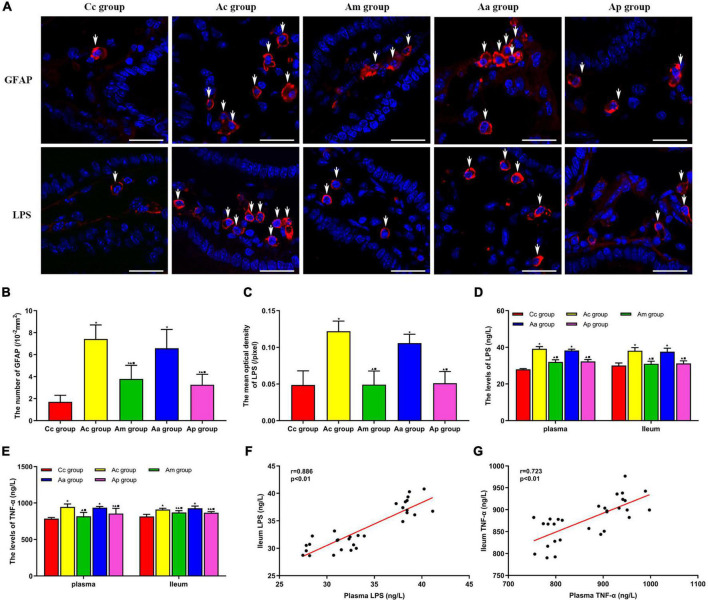
Results of the expression of GFAP, lipopolysaccharide (LPS), and TNF-α in serum and the intestine in each group (*n* = 6, mean ± SD). **(A)** Representative images of immunofluorescence (IF) staining of LPS (red) and GFAP (red) in each group, the positively stained cells are shown with white arrows, and scale bar is 25 μm. **(B)** Comparison between the number of cells expressing GFAP of all groups. **(C)** Comparison between the mean optical density of LPS of all groups. **(D,E)** Comparison between the contents of LPS and TNF-α in the serum and intestine of all groups. A one-way analysis of variance (ANOVA), followed by the LSD multiple-range test, was used with exceptions when comparing the number of cells expressing GFAP and the contents of LPS in serum, which were analyzed by the Kruskal–Wallis test. LSD-t and chi-square are presented in Supplementary Tables 5–7. ^★^*P* < 0.01 or *P* < 0.05 compared with the C57BL/6 control (Cc) group. ^▲^*P* < 0.01 or *P* < 0.05 compared with the APP/PS1 control (Ac) group; ^■^*P* < 0.01 or *P* < 0.05 compared with the APP/PS1 antibiotic + manual acupuncture (Aa) group. **(F,G)** Pearson correlation analysis between LPS and TNF-α in serum and the intestine.

The results of the expression of GFAP, LPS, and TNF-α are presented in Figure 5. GFAP was scattered in all layers of the intestine. In the Ac and Aa groups, the expression of GFAP was increased in the lamina propria of the intestine, suggesting the proliferation of EGCs, while the expression of GFAP was reduced in the Am and Ap groups. LPS is mainly found in the lamina propria of the intestine and is ovoid in shape. The fluorescence intensity of LPS in the Ac and Aa groups was higher than that in the Cc group. Compared with the Ac and Aa groups, there was a decrease in the fluorescence intensity of LPS in the Am and Ap groups.

The number of cells expressing GFAP and the mean optical density of LPS in the intestine were significantly higher in the Ac and Aa groups than in the Cc group (*P* < 0.01). Compared with the Ac and Aa groups, the number of cells expressing GFAP and the mean optical density of LPS in the Am and Ap groups were significantly decreased (*P* < 0.01).

The contents of LPS and TNF-α in the serum and intestine in the Ac and Aa groups were significantly higher than those in the Cc group (*P* < 0.01). Compared with the Ac and Aa groups, the contents of LPS and TNF-α in the serum and intestine of the Am and Ap groups were obviously decreased (*P* < 0.01 or *P* < 0.05). Pearson correlation analysis showed that there was a positive correlation between the contents of LPS in the serum and intestine of mice (*r* = 0.886, *P* < 0.01), and there was a positive correlation between the contents of TNF-α in the serum and intestine of mice (*r* = 0.723, *P* < 0.01).

## Discussion

The MWM test can effectively evaluate the spatial learning and memory ability in AD animal models and is widely used in learning- and memory-related behavioral testing and neurobiological research (Garthe and Kempermann, 2013). The results of the hidden platform trial showed that there were no significant differences in swimming speed among the groups, indicating similar group swimming abilities. The escape latency on days 2–5 and the platform crossover number of the Ac group were drastically different from those of the Cc group, demonstrating that the cognitive ability of APP/PS1 mice was significantly reduced, which was in line with the pathological changes of the AD cognitive decline (Jaroudi et al., 2017). MA and probiotics markedly reversed the aforementioned changes, indicating that both MA and probiotics could effectively improve the cognitive ability of APP/PS1 mice, which was consistent with our previous study (Chen et al., 2017; Ding et al., 2019a). Notably, the significant decline in the escape latency of the Am group occurred earlier than that of the Ap group, suggesting that the improvement in the learning ability of APP/PS1 mice induced by MA was superior to that induced by probiotics to a certain extent. To investigate the specific mechanism, MA can improve the cognitive function by regulating abnormal expressions of Aβ and Tau proteins in AD-related brain regions (Jiang et al., 2019; Sun et al., 2021), inhibiting the central inflammatory response (Ding et al., 2017), regulating cerebral blood flow (Ding et al., 2019b), modulating energy metabolism (Liu et al., 2017), and improving neuronal synaptic plasticity (Xiao et al., 2018), which exhibits the multitarget effect of MA. Beneficial metabolites and bioactive substances, such as the short-chain fatty acids (SCFAs) and neurotransmitters produced by probiotics, are transmitted to the central nervous system through the enteric nerve, spinal nerve, and vagus nerve pathways, thereby modulating the cognitive function (Bravo et al., 2011; Cryan and Dinan, 2012; Montiel-Castro et al., 2013). Therefore, we speculate that the regulation of the cognitive function by MA, which is superior to that by probiotics, may be related to the multitarget nature of MA, which deserves further study. Our behavioral results reconfirmed the benign modulating effect of MA on the cognitive function and indicated the feasibility of the MA intervention protocol in this study. The technical details used in this study, such as the relevant acupoints and operation and treatment protocols, can be useful references for future research.

Microbiota analysis found that the Sobs index of the Ac group was significantly lower than that of the Cc group, indicating that the diversity of gut microbiota in APP/PS1 mice was markedly reduced. Furthermore, the gut microbiota composition in the Ac group was also notably changed, reflected by the increase in Proteobacteria and Firmicutes and the decrease in Bacteroidetes at the phylum level and the increase in *Escherichia-Shigella* at the genus level. These results were consistent with the pathological characteristics of gut microbiota disorder in AD, which is in accordance with previous reports (Li et al., 2020; Wu et al., 2021). Compared with the Ac group, the abundance of Proteobacteria and *Escherichia-Shigella* was significantly decreased, and the abundance of Bacteroidetes was significantly increased in the Am and Ap groups. These findings confirmed, for the first time, that MA could effectively regulate the gut microbiota disorder and restore the normal microbiota structure in APP/PS1 mice, as reflected in the downregulation of Proteobacteria and *Escherichia-Shigella*, as well as the upregulation of Bacteroidetes, and the effect was comparable to that of probiotics. Previous studies have shown that acupuncture can protect the gut microbiota by promoting the growth of *Lactobacillus* and *Bifidobacterium* and inhibiting the proliferation of *Bacteroides fragilis* and *Enterococcus*, thereby exerting a therapeutic effect on many diseases such as depression and ulcerative colitis (Savari et al., 2014; Ma et al., 2019; Hong et al., 2020; Liu G. H. et al., 2020; Song et al., 2020; Wang et al., 2022). However, MA showed no significant regulation of the aforementioned flora in our study, which we speculated might be related to the existence of characteristic flora disorders in different diseases. In terms of diversity, our results indicated that MA and probiotics did not show a significant improvement in the gut microbiota diversity of APP/PS1 mice, suggesting that MA and probiotics might not play a role in regulating diversity, which is distinct from the existing research (Wang L. et al., 2020). The reason for this result may be attributable to our method of calculating diversity or our insufficient sample size. In subsequent studies, we need to increase the sample size, change the method of calculating diversity, and optimize the diversity index evaluation system for further research.

As an important part of the intestinal mucosal barrier and the brain–gut axis, the gut microbiota can participate in the establishment of the intestinal barrier and affect the function of the intestinal barrier (Camilleri et al., 2012). The increased abundance of Gram-negative bacteria often causes the excessive release of LPS (Alexandrov et al., 2019). LPS can invade the intestinal lamina propria and cause abnormal activation of EGCs, which will drive the release of proinflammatory cytokines in the enteric neuro-immune network (Kabouridis et al., 2015). Studies have found that TNF-α has a strong toxic effect on the intestinal mucosa, which can destroy the structure of TJs (West et al., 2017; Yao et al., 2019). Intestinal barrier dysfunction leads to bacterial translocation and induces systemic inflammatory reactions, which are directly related to the occurrence and development of AD (Rescigno, 2011; Pusceddu et al., 2018). Our results showed that Chiu’s score and the expression of GFAP and TNF-α in the Ac group were significantly increased, suggesting the occurrence of intestinal inflammation in APP/PS1 mice. Moreover, the loss of TJs, decreased expression of ZO-1 and occludin, and increased FITC indicated that the intestinal barrier integrity of APP/PS1 mice was destroyed and that intestinal permeability was increased. The elevated concentrations of LPS and TNF-α and positive correlations between their concentrations in serum and the intestine indicated the occurrence of gut leakage in APP/PS1 mice. The aforementioned results were in accord with the pathological features of intestinal barrier injury and intestinal inflammatory response in AD and were consistent with previous studies (Liu Q. et al., 2020; Wang L. et al., 2020). In the meantime, milder intestinal inflammation and gut leakage, as well as better barrier integrity, were observed in the Am and Ap groups. Thus, MA and probiotics can exert protective effects on the intestinal barrier and inhibitory effects on intestinal inflammation. Our study demonstrated, for the first time, that MA can effectively alleviate intestinal inflammation in APP/PS1 mice and benignly regulate the intestinal barrier function, and the effect was comparable to that of probiotics. Studies have shown that as Gram-negative bacteria, the phylum Proteobacteria contains vast opportunistic pathogens, the proliferation of which leads to a large release of LPS and induces intestinal inflammation (Chen et al., 2016). The abundance of *Escherichia-Shigella* is also positively correlated with the LPS content (Asti and Gioglio, 2014). LPS can cause an increase in intestinal permeability and intestinal barrier dysfunction by altering the normal expression of TJ proteins (He et al., 2019). Based on the gut microbiota results, we speculate that downregulating the abundance of Proteobacteria and *Escherichia-Shigella*, reducing the LPS load, and upregulating the expression of ZO-1 and occludin may be important mechanisms, by which MA reduces intestinal inflammation and benignly regulates the intestinal barrier function in APP/PS1 mice. Neuroinflammation is widely considered as a central event in the pathogenesis of AD (Finneran and Nash, 2019), and the destruction of the intestinal barrier is regarded as an essential link that leads to neuroinflammation in AD (Wang Y. et al., 2020). Gut leakage causes LPS and a large number of inflammatory mediators to enter the central nervous system, which will trigger the inflammatory response through the TLR-mediated NK-kappa B and EGFR-NF-kappa B signaling pathways (Alhasson et al., 2017; Welcome, 2019). Therefore, the gut mechanism by which MA reduces the neuroinflammation associated with AD is worth further exploration. In future, we should observe the effect of MA on neuroinflammation in APP/PS1 mice on the basis of this study so as to gain more evidence to explore and illustrate the gut mechanism of MA in the treatment of AD.

In our study, the mice in the Aa group were treated with an antibiotic mixture to disrupt the gut microbiota to determine the causal relationship between MA regulation of gut microbiota and MA regulation of the intestinal barrier function and cognitive ability. The 16S rRNA analysis showed that the abundance and uniformity of the gut microbiota in the Aa group were significantly reduced after 7 days of antibiotic pretreatment. After the subsequent MA and antibiotic drinking, the Aa group showed a more severe intestinal flora imbalance than the other four groups, indicating that antibiotic exposure led to the persistent disorder of the gut microbiota in APP/PS1 mice. At the same time, more severe cognitive deficits, intestinal inflammation, and intestinal barrier damage were observed in the Aa group. The aforementioned results indicated that in the state of persistent gut microbiota disorder, the beneficial regulation of the cognitive function and intestinal barrier function in APP/PS1 mice by MA was inhibited, demonstrating that the gut microbiota may play an important role in the beneficial regulation of the cognitive function and intestinal barrier function by MA. Related studies have shown that antibiotics can change the normal composition of the gut microbiota (Minter et al., 2016; Kurilshikov et al., 2017; Zarrinpar et al., 2018), and at the same time, antibiotics can exert intervention effects on the intestinal barrier and cognitive function, but the conclusions of relevant studies are still inconsistent (Sartor, 2004; Kountouras et al., 2009; Wang et al., 2014). Therefore, in future studies, we should establish an antibiotic-alone group and perform the MWM test after antibiotic pretreatment to further clarify whether the benign modulatory effect of MA on the cognitive function versus intestinal barrier function is achieved through the modulation of the gut microbiota.

## Conclusion

In this study, we reconfirmed that MA can beneficially regulate the cognitive function in the APP/PS1 mice and was superior to probiotics to a certain degree. We reported, for the first time, that MA can benignly modulate gut microbiota dysbiosis in the APP/PS1 mice, which manifested as the upregulation of Bacteroidetes as well as the downregulation of Proteobacteria and *Escherichia-Shigella*. We first proved that MA can effectively inhibit intestinal inflammation and alleviate intestinal barrier impairment in APP/PS1 mice, and the effect was comparable to that of probiotics. The gut microbiota may play an important role in the beneficial regulation of cognitive function and intestinal barrier function by MA. The beneficial effects of MA on the cognitive function and intestinal barrier function might be achieved through gut microbiota regulation.

## Data availability statement

The datasets presented in this study can be found in online repositories. The names of the repository/repositories and accession number(s) can be found below: https://www.ncbi.nlm.nih.gov/bioproject/PRJNA850093.

## Ethics statement

The animal study was reviewed and approved by Medicine and Animal Ethics Committee of the Beijing University of Chinese Medicine.

## Author contributions

XH performed experiments, analyzed data, and wrote the manuscript. ND conceived and supervised the project and designed the study. YZ and YY performed experiments. YLZ and JZ helped with data collection. YL helped with immunofluorescence experiments. ZL reviewed the manuscript. All authors contributed to the article and approved the submitted version.
