# Supplementary material for: Benign regulation of the gut microbiota: The possible mechanism through which the beneficial effects of manual acupuncture on cognitive ability and intestinal mucosal barrier function occur in APP/PS1 mice
Source: Front Neurosci. 2022 Aug 3;16:960026. doi: 10.3389/fnins.2022.960026 (PMC9382294; doi:10.3389/fnins.2022.960026)
Supplement: Supplementary file 1 [file Data_Sheet_1.docx]

**SUPPLEMENTARY TABLE 1** **| The LSD-t of escape latency in hidden platform trial (t, P).**

| **Groups** | **Day 2** | **Day 3** | **Day 4** | **Day 5** |
| --- | --- | --- | --- | --- |
| Cc | - | - | - | - |
| Ac | (-4.12; < 0.001) | (-5.50; < 0.001) | (-5.46; < 0.001) | (-5.94; < 0.001) |
| Am | Am-Cc (-1.84; 0.072)  Am-Ac (2.28; 0.028) | Am-Cc (-2.21; 0.032)  Am-Ac (3.29; 0.002) | Am-Ac (3.82; < 0.001) | Am-Ac (4.16; < 0.001) |
| Aa | Aa-Cc (-3.89; < 0.001)  Aa-Am (-2.05; 0.047) | Aa-Cc (-5.19; < 0.001)  Aa-Am (-2.98; 0.005)  Aa-Ap (-2.33; 0.024) | Aa-Cc (-5.01; < 0.001)  Aa-Am (-3.37; 0.002)  Aa-Ap (-2.25; 0.029) | Aa-Cc (-5.39; < 0.001)  Aa-Am (-3.61; 0.001)  Aa-Ap (-2.46; 0.018) |
| Ap | Ap-Ac (2.71; 0.009) | Ap-Cc (-2.86; 0.006)  Ap-Ac (2.63; 0.012) | Ap-Cc (-2.76; 0.008)  Ap-Ac (2.70; 0.010) | Ap-Cc (-2.93; 0.004)  Ap-Ac (3.01; 0.010) |

**SUPPLEMENTARY TABLE 2** **| The Chi-Square of the Platform crossover numbers, Chiu’s score and sobs index (Chi-Square, P).**

| **Groups** | **Platform crossover numbers** | **Chiu’s score** | **Sobs index** |
| --- | --- | --- | --- |
| Cc | - | - | - |
| Ac | (15.27; < 0.001) | (8.21; 0.002) | (6.35; 0.014) |
| Am | Am-Ac (5.99; 0.012) | Am-Ac (4.37; 0.035) | - |
| Aa | Aa-Cc (14.29; < 0.001)  Aa-Am (4.59; 0.026)  Aa-Ap (7.47; 0.025) | Aa-Cc (7.83; 0.003)  Aa-Am (3.73; 0.048)  Aa-Ap (5.43; 0.024) | Aa-Cc (11.31; < 0.001)  Aa-Ac (11.31; < 0.001)  Aa-Am (11.31; < 0.001)  Aa-Ap (11.31; < 0.001) |
| Ap | Ap-Ac (9.43; 0.012) | Ap-Ac (6.40; 0.017) | - |

**SUPPLEMENTARY TABLE 3** **| The Chi-Square of the microbial relative abundance (Chi-Square, P).**

| **Groups** | **Bacteroidota (phylum level)** | **Proteobacteria (phylum level)** | **Firmicutes (phylum level)** | **Escherichia-Shigella (genus level)** |
| --- | --- | --- | --- | --- |
| Cc | - | - |  |  |
| Ac | (9.93; < 0.001) | (6.36; < 0.01) | (3.98; < 0.01) | (4.65; < 0.05) |
| Am | Am-Ac (4.86; < 0.01) | Am-Ac (8.37; < 0.01) | Am-Ac (0.01; < 0.01) | Am-Ac (7.09; < 0.05) |
| Aa | Aa-Cc (11.65; < 0.001)  Aa-Am (11.65; < 0.001)  Aa-Ap (11.65; < 0.001) | Aa-Cc (11.36; < 0.001)  Aa-Am (11.36; < 0.001)  Aa-Ap (11.34; < 0.001) | Aa-Cc (11.53; < 0.001)  Aa-Am (11.53; < 0.001)  Aa-Ap (11.53; < 0.001) | Aa-Cc (11.46; < 0.001)  Aa-Am (12.31; < 0.001)  Aa-Ap (12.31; < 0.001) |
| Ap | Ap-Ac (4.41; < 0.05) | Ap-Ac (11.29; < 0.01) | Ap-Ac (0.09; < 0.01) | Ap-Ac (7.09; < 0.05) |

**SUPPLEMENTARY TABLE 4** **| The LSD-t of the FITC-dextran level (t, P).**

| **Groups** | **FITC-dextran level** |
| --- | --- |
| Cc | - |
| Ac | (-8.99; < 0.001) |
| Am | Am-Ac (8.18; < 0.001) |
| Aa | Aa-Cc (-11.57; < 0.001)  Aa-Am (-10.76; < 0.001)  Aa-Ap (-9.14; < 0.001) |
| Ap | AP-Cc (-2.43; 0.023)  Ap-Ac (6.56; < 0.001) |

**SUPPLEMENTARY TABLE 5** **| The LSD-t of the expression of Occludin, ZO-1 and LPS in IF (t, P).**

| **Groups** | **Occludin** | **ZO-1** | **LPS** |
| --- | --- | --- | --- |
| Cc | - | - | - |
| Ac | (14.55; < 0.001) | (14.55; < 0.001) | (-7.81; < 0.001) |
| Am | Am-Cc (7.42; < 0.001)  Am-Ac (-7.13; < 0.001) | Am-Cc (7.42; < 0.001)  Am-Ac (-7.13; < 0.001) | Am-Ac (7.76; < 0.001) |
| Aa | Aa-Cc (12.73; < 0.001)  Aa-Am (5.32; < 0.001)  Aa-Ap (3.47; 0.002) | Aa-Cc (12.73; < 0.001)  Aa-Am (5.32; < 0.001)  Aa-Ap (3.47; < 0.001) | Aa-Cc (-6.09; < 0.001)  Aa-Am (-6.04; < 0.001)  Aa-Ap (-5.82; < 0.001) |
| Ap | Ap-Cc (9.26; < 0.001)  Ap-Ac (-5.28; < 0.001) | Ap-Cc (9.26; < 0.001)  Ap-Ac (-5.28; < 0.001) | Ap-Ac (7.55; < 0.001) |

**SUPPLEMENTARY TABLE 6** **| The Chi-Square of the expression of ZO-1 in WB, the contents of LPS in the serum in ELISA and the number of cells expressing GFAP (Chi-Square, P).**

| **Groups** | **ZO-1** | **LPS in the serum** | **The number of cells expressing GFAP** |
| --- | --- | --- | --- |
| Cc | - | - | - |
| Ac | (8.31; < 0.001) | (8.40; < 0.001) | (27.44; < 0.001) |
| Am | Am-Ac (8.31; 0.011) | Am-Ac (8.34; 0.006) | Am-Cc (23.04; 0.002)  Am-Ac (23.71; < 0.001) |
| Aa | Aa-Cc (8.31; < 0.001)  Aa-Am (8.31; 0.018)  Aa-Ap (8.31; 0.014) | Aa-Cc (8.37; < 0.001)  Aa-Am (8.34; 0.022)  Aa-Ap (8.34; 0.049) | Aa-Cc (27.50; < 0.001)  Aa-Am (19.70; 0.001)  Aa-Ap (25.73; < 0.001) |
| Ap | Ap-Ac (8.31; 0.008) | Ap-Ac (8.31; 0.015) | Ap-Cc (18.33; 0.015)  Ap-Ac (26.58; < 0.001) |

**SUPPLEMENTARY TABLE 7** **| The LSD-t of the expression of Occludin in WB, the contents of LPS in the intestine and TNF-α in the serum and intestine in ELISA (t, P).**

| **Groups** | **Occludin** | **LPS in the intestine** | **TNF-α in the serum** | **TNF-α in the intestine** |
| --- | --- | --- | --- | --- |
| Cc | - | - | - | - |
| Ac | (3.91; 0.001) | (-8.84; < 0.001) | (-6.19; < 0.001) | (-6.79; < 0.001) |
| Am | Am-Ac (-2.98; 0.006) | Am-Ac (7.84; < 0.001) | Am-Ac (4.92; < 0.001) | Am-Cc (3.91; 0.001)  Am-Ac (2.88; < 0.008) |
| Aa | Aa-Cc (4.13; < 0.001)  Aa-Am (3.19; 0.004)  Aa-Ap (4.88; < 0.001) | Aa-Cc (-8.24; < 0.001)  Aa-Am (-7.24; < 0.001)  Aa-Ap (-6.99; < 0.001) | Aa-Cc (-5.79; < 0.001)  Aa-Am (-4.52; < 0.001)  Aa-Ap (-3.13; 0.004) | Aa-Cc (-8.05; < 0.001)  Aa-Am (-4.14; < 0.001)  Aa-Ap (-4.39; < 0.001) |
| Ap | Ap-Ac (-4.66; < 0.001) | Ap-Ac (7.59; < 0.001) | Ap-Cc (-2.67; 0.013)  Ap-Ac (3.53; 0.002) | Ap-Cc (-3.65; 0.001)  Ap-Ac (3.14; 0.004) |
